# Effect of Feeding System on Muscle Fiber Composition, Antioxidant Capacity, and Nutritional and Organoleptic Traits of Goat Meat

**DOI:** 10.3390/ani13010172

**Published:** 2023-01-02

**Authors:** Lu Dou, Ye Jin, Huijiao Li, Chang Liu, Zhihao Yang, Xiaoyu Chen, Lina Sun, Lihua Zhao, Lin Su

**Affiliations:** College of Food Science and Engineering, Inner Mongolia Agricultural University, Hohhot 010018, China

**Keywords:** feeding system, muscle fiber characteristics, antioxidant capacity, nutritional traits, meat quality, fatty acid compositions, flavor, goat meat

## Abstract

**Simple Summary:**

Nutritional and organoleptic traits are important components of the meat industry, which can affect consumers’ purchasing desire and the processing of meat quality. This study evaluated the effect of feeding system on the muscle fiber characteristics, antioxidant capacity, and nutritional and organoleptic traits of goat meat. It was found that the feeding system had great effect on muscle fiber composition, antioxidant capacity, nutritional value, and organoleptic traits of goat meat, but compared with grazing on mountain range (whole area with about 40% inclination), grazing on flatland (whole area with about 0% inclination) improved the meat nutritional value and quality by altering the myofiber characteristic and antioxidative status. This study assists us in comprehending the influence of diverse feeding systems on nutritional and organoleptic traits of goat meat so as to develop more appropriate measures to retain superior quality of goat meat and provide basic data for the development of goat meat products.

**Abstract:**

The objective of this study was to evaluate the effect of feeding system on muscle fiber composition, antioxidant capacity, and nutritional and organoleptic traits of goat meat. Goats that grazed on flatland (whole area with about 0% inclination, FG group) and mountain range (whole area with about 40% inclination, MG group) were selected for the analysis. The results showed that grazing on flatland increased oxidized-twitch fiber percentage, the expression of the MyHC IIa gene (*p* < 0.001), the activity of glutathione peroxidase (GSH-Px) (*p* < 0.05), total antioxidant capacity (T-AOC) (*p* = 0.001), and radical scavenging ability (RSA) (*p* < 0.05); meanwhile, the MyHC IIb gene expression (*p* < 0.01) and malondialdehyde (MDA) content (*p* = 0.001) were decreased. Feeding system affected nutritional and organoleptic traits of goat meat, and grazing on flatland increased protein content, total content of monounsaturated fatty acid (MUFA), polyunsaturated fatty acid (PUFA), pH_45min_, a* value, and variety of volatile flavor compounds but decreased the content of saturated fatty acid (SFA), shear force, and b* value. In addition, the key flavor substances were screened using relative odor activity value (ROAV), including hexanal, heptanal, (E)-2-octenal, octanal, nonanal, decanal, (E)-2-nonenal, and 1-octen-3-ol. Among them, 1-octen-3-ol and (E)-2-nonenal were the most contributing flavor compounds in the FG and MG groups, respectively, providing distinctive odor to goat meat.

## 1. Introduction

In recent years, meat nutritional value and quality have been considered critical parameters of consumer interest [1], which can be affected by diet [2], feeding system [3], sex [4], et cetera. Among them, feeding system is crucial for the accumulation of nutrients and the formation of meat quality in animals. Numerous studies have confirmed that differences in feeding system could be attributed to diet, exercise, and environment (altitude), which affect muscle metabolism and ultimately impact nutritional and organoleptic traits of meat. Wang et al. [5] found that meat quality, fatty acid composition, and metabolism were affected extremely by feeding regimens, and further analysis revealed that this could be attributed to differences in exercise and diet. Hou et al. [6] reported that exercise of Mongolia sheep between two feeding regimens differed, which resulted in different meat quality and muscle fiber types. Another study revealed that fatty acid composition of calves from three different altitudes, namely lowlands (< 400 m), ridges (400–700 m), and mountains (>700 m) differed significantly [7].

Muscle fibers are the basic constituents of skeletal muscle and could be divided into different types. A report showed that meat quality and fatty acid composition could both be influenced by the conversion of muscle fiber types [8]. Joo et al. (2017) found that the intramuscular fat (IMF) content and the proportions of saturated fatty acid (SFA) and monounsaturated fatty acid (MUFA) were correlated with types of muscle fibers, including the longissimus lumborum muscle, in Hanwoo steers [9]. In addition, Dou et al. (2022) studied the relationship between oxidative stability and the flavor of different muscle parts of lamb, and they found that oxidized muscle fibers showed a higher antioxidant capacity, which affected the formation of flavor substances [10]. Importantly, the type of muscle fibers is also impacted by feeding system. A previous study showed that pasture feeding regimen can lead to an increased proportion of oxidized muscle fibers compared to confinement feeding regimen [6].

The antioxidant system can prevent the initiation of lipid chain reactions or eliminate the free radicals that have been initiated to maintain the oxidative stability of meat. Malondialdehyde (MDA) is an important marker of the degree of lipid oxidation. Antioxidant enzymes are an important indicator of the antioxidant status of tissues, of which superoxide dismutase (SOD), catalase (CAT), and glutathione peroxidase (GSH-Px) are three key antioxidant enzymes that are essential in the antioxidant system. Previous studies demonstrated that oxidative stability was affected by feeding regimens. Luo et al. [11] reported that grazing sheep revealed higher antioxidant capacity due to the long-term consumption of fresh pasture compared to captive sheep. In addition, the muscle antioxidant capacity was closely related to meat quality. For example, the antioxidant capacity of muscles can improve meat color stability by enhancing the myoglobin resistance to oxidation [12].

The Erlangshan white velvet goat is an autochthonous breed in China, mainly distributed in Inner Mongolia Urad Grassland (located around 41°34´N, 108°31´E), which has been defined as an excellent breed for both meat and fleece. A long-term grazing feeding system without any supplementary feeding makes goat meat considered to be of superior quality; however, there are no available data on whether differences in feeding system affect the nutritional and organoleptic quality of goat meat. Accordingly, there was a hypothesis that feeding regimen (grazing on mountain versus flatland) may regulate antioxidant capacity and muscle fiber characteristics and then alter nutritional and organoleptic traits. The aim of the study was to evaluate the effect of feeding system on muscle fiber, antioxidant capacity, nutritional value (chemical composition and fatty acid composition), and organoleptic traits (pH, color, cooking loss, shear force, and volatile flavor compounds) of goat meat and explain the differences on meat nutritional value and organoleptic quality from the perspective of muscle fibers and antioxidant capacity.

## 2. Materials and Methods

### 2.1. Animals and Experimental Design

Animal experiments were carried out approved by the Ethics Committee of Inner Mongolia Agricultural University (permit number NND2021072). A total of 18 (9 FG group and 9 MG group, initial body weight: FG group = 51.74 ± 6.03 kg, MG group = 51.47 ± 5.93, *p* = 0.937) normally developed and disease-free adult male goats aged approximately 5 years old were selected from two areas (whole area with about 0% inclination, FG group; whole area with about 40% inclination, MG group) in Bayan Nur City, Inner Mongolia Autonomous Region, China. The selection of goats for analysis was based on age and live weight to stem the effect of the growth rate. Data related to the natural conditions were collected from Urad Central Banner People’s Government, and the categories of forage grass were obtained from the research of Zhao [13] (shown in Table 1). Goats diet was natural pasture without supplemented with concentrates, and the conventional nutrient composition of mixed forage grass is shown in Table 2. The animals were transported to a commercial slaughterhouse (32 km) and humanely slaughtered on the same day. All procedures were conducted in accordance with Committee of Animal Experimentation.

### 2.2. Sample Collection

After slaughtering, longissimus thoracis (LT) samples from the left sides of the carcasses (from the 10th rib to the 13th rib) were stored at 4 °C for meat quality analysis. Then, 10 g sample of LT muscle was snap-frozen in liquid nitrogen until analysis. In addition, the LT sample (0.5 × 0.5 × 1 cm) was cryofixed in isopentane cooled for histochemical analysis. Meanwhile, about a 50 g sample was taken and stored at −20 °C.

### 2.3. Histochemical Analysis

Next, 10 μm flakes were obtained by cryomicrotome, and myosin ATP staining to was performed classify the muscle fibers [14]. About 1500 fibers were used to analyze the diameter, cross-sectional area, fiber number composition, and fiber area composition.

### 2.4. Real-Time Quantitative PCR

Trizol reagent (TaKaRa, Dalian, China) was used to extract the RNA; then, a PrimeScript RT reagent kit (TaKaRa, Dalian, China) was used to reverse-transcribe the total RNA into cDNA. The thermal cycling conditions were set on the PCR machine (LightCycler®96, Basel, Switzerland) as follows: 95 °C for 30 s, 35 cycles of 95 °C for 5 s, 60 °C for 30 s, and 72 °C for 30 s. The primer sequences are shown in Table 3. The gene expression was calculated according to the method reported by Livak et al. [15].

### 2.5. Oxidative Stability Measurement

Antioxidant capacity analysis of meat samples was performed using kits obtained from Nanjing Jiancheng (Nanjing, Jiangsu, China). Malondialdehyde (MDA) content, activities of superoxide dismutase (SOD), catalase (CAT), glutathione peroxidase (GSH-PX), and total antioxidant capacity (T-AOC) were measured using MDA assay kit (TBA method: No. A003-1-1), SOD assay kit (WST-1 method: No. A001-3-2), CAT assay kit (colorimetric method: No. A007-2-1), GSH-PX assay kit (colorimetric method: No. A005-1-1), and T-AOC assay kit (colorimetric method: No. A015-1-2), respectively.

### 2.6. Determination of Meat Nutrition

The content of protein, moisture, ash, and fat of goat meat were determined according to GB 5009.5-2016 (Kjeldahl method), GB 5009.3-2016 (high-temperature drying method), GB 5009.4-2016 (muffle cauterization method), and GB 5009.6-2016 (Soxhlet extraction method), respectively.

### 2.7. Determination of Fatty Acids

The fatty acids were extracted in accordance with the method described by Folch et al. (1957) [16]. After extraction, the fatty acid composition was analyzed using a GC (PE Clarus680) equipped with an SP2560 capillary column (length: 100 m; internal diameter: 0.25 mm; film thickness: 0.20 μm; Restek). Nitrogen (1.1 ml/min) was used as the carrier gas. The gas chromatography protocol was as follows: the oven temperature was held at 120 °C for 5 min, then increased to 230 °C (3 °C/min) and subsequently to 240 °C (1.5 °C/min) for 13 min. The temperature of the detector and injector were held at 260 °C. The fatty acid was identified by comparison with the standard fatty acid mixture (37-component FAME mixture, CRM47885, Sigma, Saint Louis, MO, USA). Finally, the relative contents of the fatty acid were calculated.

### 2.8. Determination of Meat Quality

The pH_45min_ and pH_24h_ were assessed using portable pH meter (SenvenGo, Mettler Toledo, Switzerland). Before measurements, the pH meter was adjusted in buffers (pH 4.60 and 7.00) at 4 °C. The meat color (L*, a*, b*) was assayed using a CR-410 chromometer (Konica Minolta, Japan), the chromometer was calibrated with a standardized white tile, at 2° observer angle, 50 mm aperture size and the illuminant D65. For the cooking loss and shear force value measurements, LT samples were weighed and packaged in polythene bags and heated in water bath (80 °C) until the temperature of meat reached 70 °C. After cooling to room temperature, the samples were then removed and reweighed to calculate the cooking loss. Then, the sample was cut into 3 × 1 × 1 cm strip. The shear force was measured using tenderness meter (LM3, Northeast Agricultural University, Harbin, China). 

### 2.9. Determination of Volatile Flavor Compounds

Volatile flavor compounds were extracted according to Vasta et al. [17]. Briefly, a muscle sample (5 g) was placed in a 15 mL vial, and a solid-phase microextraction fiber (Supelco, Bellefonte, USA) was exposed over the sample and extracted at 60 °C for 40 min. Then, it was desorbed for 3 min. The gas chromatograph (TRACE 1300, Thermo Fisher Scientific, Waltham, USA) settings were as follows: the oven temperature was held at 40 °C for 5 min, increased to 200 °C (5 °C/min), and then ramped at a rate of 20 °C/min to 250 °C. The mass spectra were acquired at 70 eV with a scan range of 30 to 400 m/z. The flavor substances were identified using the NIST MS Search 2.0 database. The relative contents of volatiles were analyzed using the area normalization method.

### 2.10. Relative Odor Activity Value (ROAV) Analysis of Volatile Components

ROAV analysis was used to assess the contribution of volatile compounds to the meat flavor [18].

ROAVi ≈ 100 × C%i/C%s × Ts/Ti(1)

Ci and Ti represent the relative contents of the flavor compounds and their thresholds, respectively; Cs and Ts represent the relative contents of dominant components contributing to the meat flavor and their thresholds, respectively. An ROAV > 1 is regarded as the key flavor compound, and an ROAV between 0.1 and 1 is considered a flavor modifier.

### 2.11. Statistical Analysis

All the experimental data were arranged in Excel 2019 and analyzed using the SPSS 22.0 software. One-way analysis of variance (ANOVA) was used to compare the results of the muscle fiber, antioxidant capacity, meat nutritional value, and organoleptic traits of the two groups. All the data were expressed as the means ± SEM. *p* < 0.05 is considered significant.

## 3. Results

### 3.1. Muscle Fiber Characteristics

**Figure 1:**
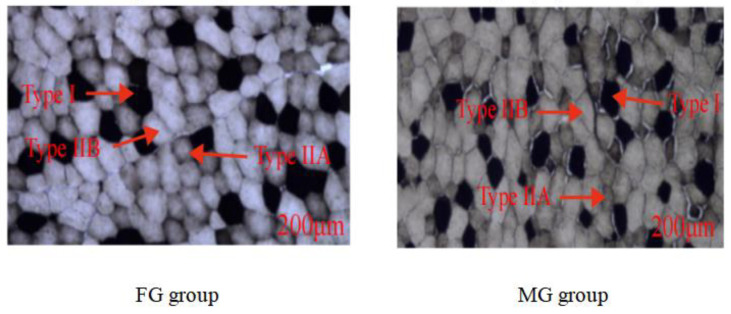
Serial sections of LT muscle stained with ATPase. FG group, flatland-grazing group; MG group, mountain-grazing group; bar: 200 μm.

**Figure 2:**
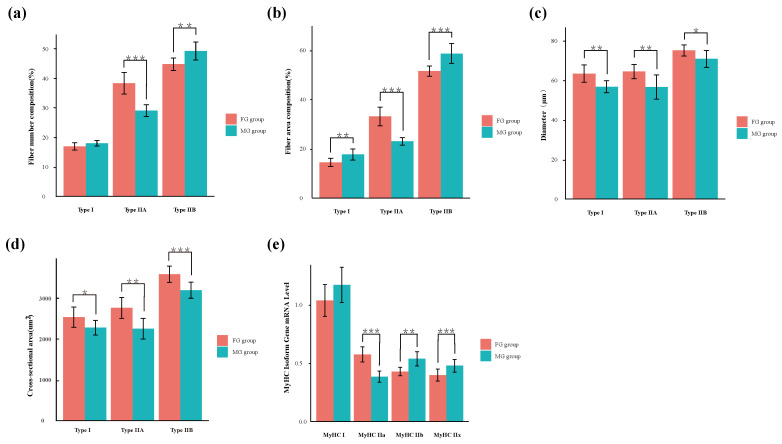
Differences in muscle fiber characteristics and myosin heavy-chain (MyHC) isoform gene mRNA levels of LT muscle of goats in two groups. (**a**) Fiber number composition; (**b**) fiber area composition; (**c**) muscle fiber diameter; (**d**) cross-sectional area of muscle fibers; (**e**) MyHC isoform gene mRNA level. FG group, flatland-grazing group; MG group, mountain-grazing group. Levels of significance: * significant at *p* < 0.05; ** significant at *p* < 0.01; *** significant at *p* < 0.001.

The photomicrographs of myosin ATPase staining were shown in Figure 1, and the muscle fiber characteristics and the mRNA level of the MyHC isoform gene were shown in Figure 2. As shown in Figure 1, the muscle fibers were divided into three types including type I (slow oxidation, black), type IIA (fast oxidation, white), and type IIB (fast glycolysis, brown). In addition, the proportion of type IIA muscle fibers (Figure 2a, *p* < 0.001) and the area ratio type IIA muscle fibers (Figure 2b, *p* < 0.001) in the FG group were significantly higher than those in the MG group, while the proportion of type IIB muscle fibers (Figure 2a, *p* = 0.002), the area ratios of type I (Figure 2b, *p* = 0.002) and IIB muscle fibers (Figure 2b, *p* < 0.001) in the FG group were significantly lower than those in the MG group. In addition, the diameters and cross-sectional areas of the three types of muscle fiber (type I, IIA, and IIB) in the FG group were all significantly higher than those in the MG group (*p* < 0.05).

As shown in Figure 2e, the mRNA level of the MyHC IIa isoform gene in the FG group was significantly higher than that in the MG group (*p* < 0.001); however, the level of MyHC IIb was significantly lower than that in the MG group (*p* = 0.009).

### 3.2. Oxidative Stability

**Figure 3:**
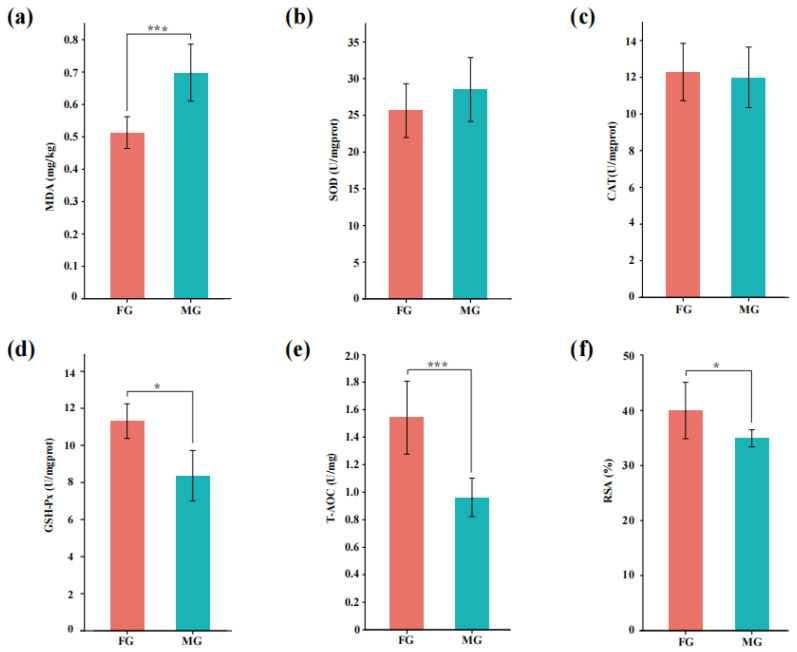
Antioxidant capacity of LT muscle of goats in two groups. (**a**) MDA content; (**b**–**d**) the activity of SOD, CAT, and GSH-Px; (**e**) the capacity of T-AOC; (**f**) the ability of radical scavenging. FG group, flatland-grazing group; MG group, mountain-grazing group; levels of significance: * significant at *p* < 0.05; *** significant at *p* < 0.001.

As shown in Figure 3a, the content of MDA of LT muscle of goats in the FG group was significantly lower than that in the MG group (*p* = 0.001), and the activity of GSH-Px (*p* = 0.019) and capacity of T-AOC (*p* = 0.001) as well as the RSA (*p* = 0.049) value of the FG group were all significantly higher than the MG group.

### 3.3. Nutritional Traits

The effect of feeding system on nutritional traits is shown in Table 4. The protein content in the FG group was significantly higher than the MG group (*p* < 0.05). However, no significant differences were discovered among meat from the two feeding systems in moisture, ash, and fat content (*p* > 0.05).

### 3.4. Fatty Acid Composition

As can be observed in Table 5, the content of myristic (C14:0) (*p* = 0.012) and margaric (C17:0) (*p* = 0.017), oleic acid (C18:1 n9c) (*p* = 0.044), linoleic acid (C18:2 n6c) (*p* < 0.001), MUFA (*p* = 0.003), PUFA (*p* = 0.005), the ratio of n-6:n-3 (*p* < 0.001), and P:S (*p* = 0.027) of goat meat in the FG group were significantly higher than those in the MG group, while the content of palmitic acid (C16:0) (*p* < 0.001) and SFA (*p* = 0.037) was significantly lower than the MG group. Trans-linoleic acid (C18:2n6t) was only detected in the FG group.

### 3.5. Meat Quality

As shown in Table 6, b* (*p* = 0.009) and shear force values (*p* = 0.030) of the LT muscle in the FG group were significantly lower than MG group, while the pH_45min_ (*p* = 0.022) and a* (*p* = 0.002) values were significantly higher than in the MG group.

### 3.6. Volatile Flavor Compound

**Figure 4:**
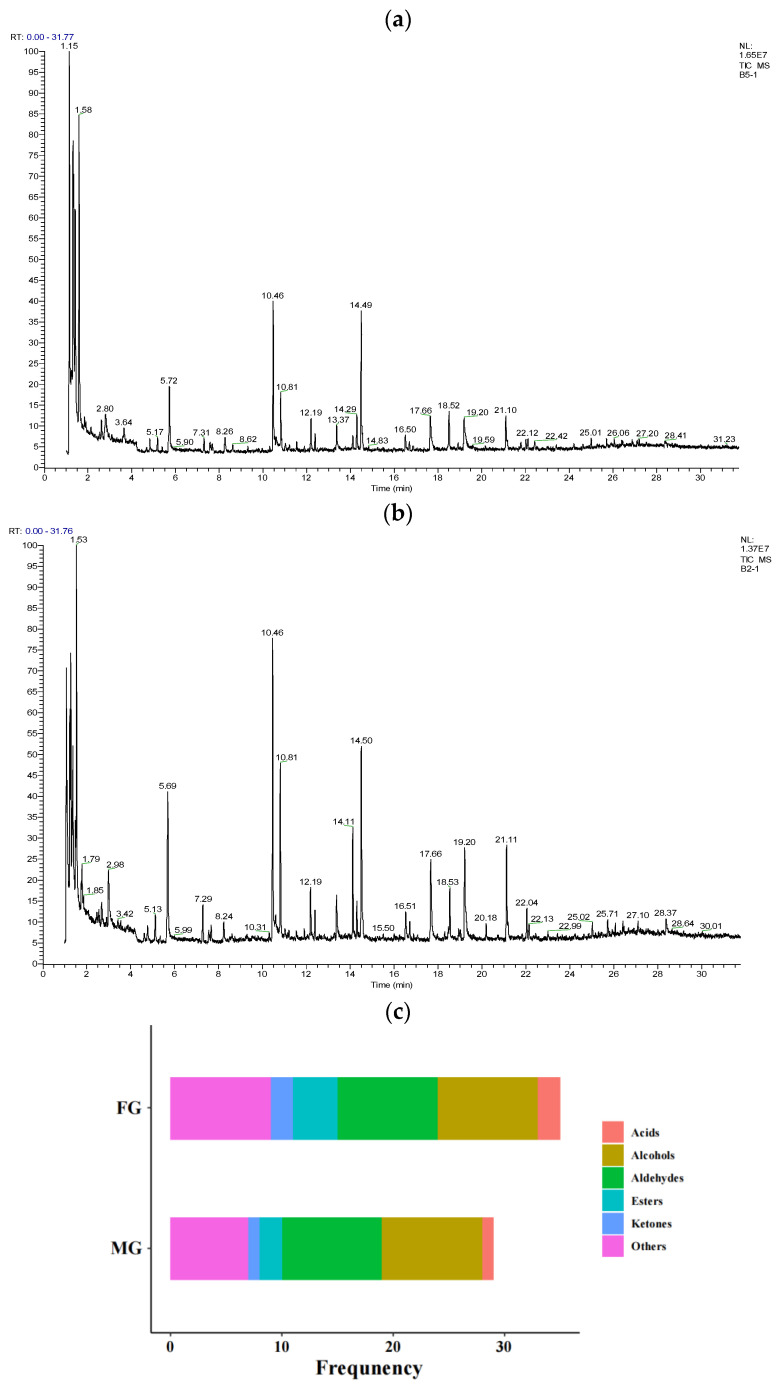
(**a**,**b**) Chromatogram of volatile flavor compounds. (**a**) FG group. (**b**) MG group. (**c**) Species of volatile flavor substances. FG group, flatland-grazing group; MG group, mountain-grazing group.

A total of 29 volatile flavor compounds in the MG group and 35 in the FG group were detected (Figure 4). Overall, the number of volatile flavor compounds in the FG group was higher than that in the MG group.

As shown in Table 7, the relative contents of hexanal (*p* = 0.009), octanal (*p* = 0.040), and butanal-3-methyl (*p* = 0.023) were lower in the FG group than the MG group. Hexanal, heptanal, (E)-2-octenal, octanal, nonanal, decanal, and (E)-2-nonenal were defined as the critical flavor substances in two groups (ROAV > 1), and (E)-2-nonenal was defined as the most contributing volatile flavor compound (ROAV = 100) in the MG group (Table 8). Alcohols were also analyzed in this study, and as shown in Table 7, the relative contents of 1-octen-3-ol (*p* = 0.006) and 2-hexadecanol (*p* = 0.049) in the FG group were significantly lower than those in the MG group. 1-octanol was identified as the volatile flavor compounds modifier for the meat in both groups (0.1 < ROAV < 1), while 1-octen-3-ol was detected in both groups and had the highest ROAV in the FG group (ROAV = 100). Moreover, feeding system had no effect on acids, ketones, esters, and other flavor substances (except toluene).

## 4. Discussion

In this study, the muscle fiber types and MyHC mRNA levels were measured to estimate the muscle fiber characteristics. The muscle fibers were divided into three types, including type I (black), type IA (white), and type IIB (brown) according to Brooke et al. [14], and combined with fiber number composition, it was found that the type IIB muscle fibers were dominant in the LT muscle of goats under two grazing systems, reaching 44.8% (FG group) and 49.3% (MG group), respectively. In addition, the higher proportion of type IIA muscle fibers and lower proportion of type IIB muscle fibers was consistent with the study of Gangnat et al. [19], who found that calves grazed on steep slopes showed a higher proportion of type IIB muscle fiber at the expense of the type IIA muscle fiber compared to calves grazed on flat pastures. On the whole, the FG group showed higher proportion of oxidized muscle (type I + type IIA) fibers (FG group = 55.3%, MG group = 47.1%), suggesting that the FG group had more oxidized muscle fibers than the MG group. Notably, the diameters and cross-sectional areas of the three types of muscle fiber (type I, IIA, and IIB) in the FG group were elevated by grazing on flatland. A previous study demonstrated that diet affected the transformation of muscle fibers remarkably, and dietary antioxidants supplementation could increase mitochondrial biogenesis and the ratio of oxidized muscle fibers [20]. Hence, the differences in the muscle fiber types between the two groups may be related to the differences in the ingested herbage types. As shown in Table 1 and Table 2, significant differences in the types of forage grass were observed, and the mixed pastures of flatland contained more antioxidant components. Furthermore, the projection coverage of grass in the flatland was 15–25% (MG group: 8–15%), and the height of grass was 8–18 cm (MG group: 4–10 cm) [13], which may have affected the nutrient intake of the goats, eventually contributing to the high proportion of oxidized muscle fibers. Furthermore, research has shown that exercise training was an effective stimulus to activate mitochondrial function, and moderate exercise could elevate composition of oxidized muscle fibers [21], but excessive exercise training caused mitochondrial functional impairment [22]. Therefore, the exercise training of flatland-grazing goats may be more suitable. Four different MyHC isoforms have been identified in mammalian skeletal muscle [23]. As expected, an increased mRNA level of the MyHC IIa isoform gene and decreased MyHC IIb of the FG group were observed, which indicated that grazing on flatland increased the composition of type IIa fiber and decreased the type IIb fiber. Long-term intake of antioxidant pasture may be the reason for the high expression of MyHC IIa. Therefore, it might be concluded that grazing on flatland could lead to the conversion of MyHC IIb to MyHC IIa in the LT muscle of goat meat.

MDA is an index of lipid oxidation that affects consumer acceptance. The content of MDA in the LT muscle of goats in the FG group was decreased, indicating that the degree of lipid oxidation of goat meat in the MG group was higher than FG group, which may have been due to differences in the composition of the forage under the two feeding systems. Similar results were reported by Luo et al. [11], who speculated the lower MDA value in the grazing group may be attributed to the different forages compared with captive feeding. SOD is the first line of defense in the antioxidant system [24]. CAT is a peroxidase that catalyzes the decomposition of H_2_O_2_ into H_2_O and O_2_ [25]. GSH-Px inhibits further oxidative damage [26]. T-AOC is an aggregate indicator of the overall antioxidant capability [27]. The radical scavenging ability (RSA) can reflect the ability of the muscle antioxidant system to scavenge free radicals [28]. Elevated activity of GSH-Px and capacity of T-AOC as well as the RSA value were all detected in the FG group. As Nam and Ahn [29] reported, the diets of animals influenced the meat antioxidant ability deeply, and green pasture was a good resource for antioxidants. For instance, the dietary management of antioxidant substances was proven to alter the antioxidant ability of meat [30]. Overall, the antioxidant capacity of the FG group was higher than that of the MG group, and this may be due to the different pastures consumed by goats under the two feeding systems, coupled with an appropriate amount of exercise, which promoted an increase in the antioxidant capacity of the goat meat.

The effect of feeding system on nutritional traits were evaluated. The results revealed grazing on flatland increased the protein content. However, the content of moisture and ash were not affected by feeding system. Hou et al. [6] reported that moisture and ash contents were usually highly homogeneous under different feeding systems (pasture feeding regimen and confinement feeding regimen). Panjono et al. [31] also found that raising altitudes did not affect the moisture and ash content of cattle. Additionally, the fat content tended to be increased by grazing on flatland. Hu et al. [32] found that muscle with a higher mRNA expression of MyHC IIa had a higher fat content in contrast to muscle with a higher expression of MyHC IIb mRNA. Therefore, the high fat content in the FG group may have been due to the high expression of MyHC IIa.

The fatty acid compositions of the LT muscle from the two groups are thus summarized. As can be observed, the proportions of SFA, MUFA, and PUFA were different in the two groups due to differences in the majority of fatty acids, including myristic (C14:0), palmitic acid (C16:0), margaric (C17:0), oleic acid (C18:1 n9t), trans-oleic acid (C18:1 n9c), eicosenoic acid (C20:1), and linoleic acid (C18:2n6c). SFA can increase the risk of cardiovascular disease, especially coronary atherosclerosis, by increasing low-density lipoprotein (LDL) and high-density lipoprotein (HDL) in human blood [33]. In the present study, total SFA content in the MG group was higher than the FG group. Cividini et al. [34] also found that feeding regimens changed the content of SFA in the muscle tissue of lamb, which may be associated with the intake energy of lambs. MUFA is beneficial for human health and can have the function of lowering cholesterol and preventing atherosclerosis, of which oleic acid (C18:1 n9c) is the most representative fatty acid [35]. PUFA can regulate the body’s lipid metabolism and treat and prevent cardiovascular and cerebrovascular diseases [36]. Linoleic acid (C18:2 n6c) is an essential fatty acid for human body. The ratio of n-6:n-3 is an indicator of the nutritional value of meat: the closer the ratio is to (4~6):1, the higher the nutritional value of the meat [37]. The results revealed that grazing on flatland increased the contents of oleic acid (C18:1 n9c), linoleic acid (C18:2 n6c), MUFA and PUFA, the ratio of n-6: n-3, and the ratio of P:S of goat meat. Overall, compared to MG group, goat meat from the FG group showed higher nutritional value. Interestingly, trans-linoleic acid (C18:2n6t) was only detected in the FG group, which could be in part due to the differences in diet. The effect of feeding regimen on the fatty acid composition and content was also noticed by Adnoy et al. [38], who found that lambs in lowland tended to have higher MUFA contents than those grazed in mountains. Consequently, the difference in grazing regimen also affected the fatty acid composition of the goat meat. Differences in the botanical diversity and availability of the pasture between altitudes could influence the fermentation processes of substances in the rumen, contributing to the differences in the fatty acid composition of the meat [39]. It was demonstrated that foraged legumes may modify the fatty acid profile in meat [38]. The Caragana shrub, as a typical legume plant, is widely distributed in flatland areas [13], which may affect the fatty acid composition of goat meat. Focusing on rabbit, Alasnier et al. [40] reported that oxidative muscles fibers were accompanied by lower PUFA contents. Leseigneur et al. [41] found that SFA and MUFA were not affected by muscle fiber type, which is different from the present results. This difference between cattle and goat muscles may be due to the energy metabolism and contractile properties. 

Next, the differences between the two groups in meat quality were evaluated. The pH level is a vital index affecting the rate of muscle glycolysis [42]. In the present study, grazing regimen failed to affect the pH_24h_ of muscle; however, grazing on unimproved mountain range decreased the pH_45min_ of the LT muscle. According to Rekiel et al. [43], the proportion of glycolytic fiber was negatively correlated with the pH value. As reported, a high antioxidant capacity could enhance the stability of cell membranes, resulting in a decrease in glycolytic potential [44], which led to differences in the pH value in the present study. The visual appearance of meat is important to evaluate meat quality and make purchasing decisions. b* value was decreased, while a* value was increased by feeding on flatland. Combining the results of muscle fiber, the high a* and low b* values in the FG group may be attributed to the high proportion of oxidized muscle fibers and lower proportion of glycolytic muscle fibers [6]. Notably, the a* values in both the FG (16.53) and MG (17.85) groups were >14.5, suggesting 95% confidence for consumer acceptability [36]. In addition, the cooking loss was also decreased slight by grazing on flatland, which may be associated with the decreased type IIb muscle fiber. As previously reported, the proportion of type IIb fiber was positively related to cooking loss and negatively correlated with the water-holding capacity [7]. Furthermore, Meng et al. [20] reported that decreased MDA and an increased antioxidant status may have been important aspects for the lower cooking loss of muscle, which was consistent with the present result that a low MDA level and high GSH-Px and T-AOC activity contributed to a better water-holding capacity. Meanwhile, better tenderness of goat meat in the FG group was observed, which may have been due to the elevated physical activity. Gangnat et al. [19] found that an increase in exercise can affect the metabolism of beef cattle and induce changes in the histological characteristics of muscle fibers, causing the tenderness of meat to deteriorate. For the present study, this meant that the physical activity of the MG group compared to the FG group could have been at a higher intensity or repeated more often or both because of the 40% inclination. Moreover, research has demonstrated that the proportion of oxidized myofibers in the muscle was negatively correlated with the shear force values. In the present study, the FG group had a higher proportion of oxidized myofibers that was therefore accompanied by low shear force. In conclusion, the meat quality of the LT muscle was improved by grazing on flatland, which is in line with the simultaneously enhanced antioxidant capacity of muscles, increased type IIA muscle fiber, and decreased type IIB muscle fiber. 

Volatile flavor compounds are important factors affecting the sensory attributes of meat. It was found that grazing regimen greatly affected the number of volatile flavor compounds, as evidenced by the enriched variety of volatile flavor compounds. To further analyze the effect of the grazing regime on the volatile flavor compounds of goat meat, the relative content and ROAV values were calculated for each volatile flavor compound. Aldehydes are produced by lipid oxidation and amino acid degradation [45], which is essential for meat volatile flavor compounds. In the present study, hexanal, heptanal, (E)-2-octenal, octanal, nonanal, decanal, and (E)-2-nonenal were identified as the key volatile flavor compounds in the two groups, providing the unique flavor of goat meat. Interestingly, (E)-2-nonenal was defined as the most contributing volatile flavor compound in the MG group due to its lower threshold and higher content. Notably, the relative contents of hexanal, octanal, and butanal-3-methyl were decreased by feeding on flatland. Hexanal and octanal are the oxidation products of unsaturated fatty acids. It was found that the MG group displayed higher contents of hexanal and octanal and a lower antioxidant capacity, indicating that grazing on flatland may have reduced the level of oxidation of the unsaturated fatty acids by improving the antioxidant capacity of the goat meat. Studies have shown that lower antioxidant enzyme activity may be significantly correlated with a high content of volatile aldehydes [46]. Luo et al. [11] reported that the aldehyde content of grazing-fed lambs was lower than that of captive-fed lambs due to the higher antioxidant capacity. Alcohols are also reported to participate in the formation of flavor. Grazing on mountain range elevated the relative contents of 1-pentanol, 1-octanol, 1-heptanol, and 1-hexanol-2-ethyl; however, the relative contents of 1-octen-3-ol and 2-hexadecanol were decreased. 1-octanol could impart fatty, waxy, oily, walnut, and burnt odor to goat meat and was identified as the volatile flavor compound modifier for the meat in both groups. 1-octen-3-ol, derived from linoleic acid and arachidonic acid, was detected in both groups and had the highest ROAV in the FG group, imparting to the meat a mushroom and smoke aroma.

## 5. Conclusions

The present study showed that grazing on flatland increased type IIA myofiber, enhanced oxidative status, and decreased type IIB myofiber. Meanwhile, grazing on flatland increased the nutritional value of goat meat, as evidenced by the higher protein content, total MUFA and PUFA content, and lower SFA content. Goat meat from the FG group revealed better sensory characteristics, pH_45min_, and a* value, and the variety of volatile compounds were increased, while b* and shear force values were decreased. Additionally, hexanal, heptanal, (E)-2-octenal, octanal, nonanal, decanal, (E)-2-nonenal, and 1-octen-3-ol were identified as key volatile flavor compounds, providing grassy, jasmine, mint, wet ground, bitter, coffee, citrus-like, nutty, fatty, rose, orange, lemon, wax, and mushroom odor for goat meat. This study assists us in comprehending the influence of diverse feeding systems on nutritional and organoleptic traits of goat meat so as to develop more appropriate measures to retain superior quality of goat meat and provide basic data for the development of goat meat products.

## Figures and Tables

**Table 1:** Climatic characteristics of two areas in Bayan Nur City, Inner Mongolia Autonomous Region.

Group	Altitude (m)	Daily Mean Temperature (°C)	Mean Relative Humidity (%)	Mean Wind Speed (m/s)	Types of Forage Grass
FG	1020	8.2	47.1	3.2	*Caragana interdia, Caragana stennophylla, Caragana brachypoda, Caragana intermedia, Caryopteris mongolica, Eurotia ceratoibes, Artemisiaordosica, Stipa gobica, Artemisia frigida, Cleistogenes mutica, Cleistogenes squarrosa, Ajania achilleoides, Agropyron desertorum, Aneurolepidium dasystachys*
MG	1590	2.0	58.6	5.4	*Caragana stennophylla, Stipa gobica, Convolvulus ammannii, Cleistogenes mutica, Cleistogenes squarrosa*

FG group, flatland-grazing group; MG group, mountain-grazing group.

**Table 2:** The conventional nutrient composition and content of mixed forage grass (calculated in DM).

Group	Crude Protein (%)	Crude Fat (%)	Carbohydrate (%)	High Digestible Fiber (%)	Low Digestible Fiber (%)	Lignin (%)	α-Tocopherol (mg/kg)	β-Carotene (mg/kg)
FG	12.7	3.5	19.1	10.7	50.2	7.9	13.2	450.8
MG	13.1	2.8	23.5	12.8	48.8	7.2	8.4	402.1

FG group, flatland-grazed group; MG group, mountain-grazed group; DM, dry matter.

**Table 3:** Primers used for real-time quantitative PCR.

Gene	Primer Sequence (5′-3′)	Product Length, bp	Genbank No.
*GAPDH*	F: CGGCACAGTCAAGGCAGAGAAC	115	XM_027961471.2
	R: CACGTACTCAGCACCAGCATCAC		
*MyHC* *I*	F: CAACCTGGCTGATGCGGAAGAG	111	XM_004010325.4
	R: TTCATCTCCTCCTCGTCCTCCAAC		
*MyHC* *IIa*	F: ACAGTACGAGGAGGAGCAGGAATC	106	XM_027974884.2
	R: GATGGCGTCCGTCTCATACTTGG		
*MyHC* *IIb*	F: GACATCACGCAAATCCAGGGAGAG	105	XM_027974883.2
	R: CTCAGCCATCATAGCCGCATCAG		
*MyHC* *IIx*	F: TTCCAGAAACCCAAACCTGCCAAG	101	XM_027974882.2
	R: TTGTCCAGCCAGCCAGTAATGTTG		

**Table 4:** Nutritional traits of longissimus thoracis (LT) muscle of goats in two groups.

Items	FG Group	MG Group	SEM	*p*-Value
Moisture (%)	71.55	72.70	0.39	0.148
Ash (%)	1.16	1.05	0.03	0.188
Protein (%)	22.07	20.02	0.38	0.001
Fat (%)	5.72	4.19	0.39	0.068

FG group, flatland-grazing group; MG group, mountain-grazing group; SEM, Standard error of mean.

**Table 5:** Fatty acid composition of LT muscle of goats in two groups.

Fatty Acids (%)	FG Group	MG Group	SEM	*p*-Value
C14:0	2.11	1.79	0.07	0.012
C15:0	0.32	0.28	0.03	0.577
C16:0	19.17	27.92	1.06	<0.001
C17:0	1.07	0.90	0.04	0.017
C18:0	16.40	16.03	0.41	0.677
C24:0	0.66	0.93	0.08	0.156
C16:1	1.37	1.44	0.06	0.629
C17:1	0.71	0.85	0.04	0.099
C18:1 n9t	2.70	0.99	0.28	<0.001
C18:1 n9c	46.14	41.89	1.12	0.044
C20:1	1.03	1.37	0.08	0.015
C18:2 n6t	0.13	ND	-	NS
C18:2 n6c	5.51	2.71	0.19	<0.001
C18:3 n-3	0.60	0.62	0.03	0.834
C20:3 n-3	2.08	2.28	0.15	0.610
Ʃ SFA	39.73	47.85	1.14	0.037
Ʃ MUFA	51.95	46.54	0.97	0.003
Ʃ PUFA	8.32	5.61	0.70	0.005
n-6:n-3	2.10	0.93	0.26	<0.001
P:S	0.21	0.12	0.02	0.027

Ʃ SFA, saturated fatty acid; Ʃ MUFA, monounsaturated fatty acid; Ʃ PUFA, polyunsaturated fatty acid; IMF, intramuscular fat; FG group, flatland-grazing group; MG group, mountain-grazing group; ND, not detected. P:S, Ʃ PUFA/Ʃ SFA.

**Table 6:** Meat quality traits of LT muscle of goats in two groups.

Items	FG Group	MG Group	SEM	*p*-Value
pH_45min_	6.94	6.71	0.05	0.022
pH_24h_	5.65	5.66	0.05	0.961
L*	30.71	32.02	0.45	0.153
a*	17.86	16.53	0.23	0.002
b*	2.91	3.48	0.12	0.009
Shear force (N)	77.14	89.560	2.95	0.030
Cooking loss (%)	35.49	36.80	0.83	0.463

L*, lightness; a*, redness; b*, yellowness; N, Newton; FG group, flatland-grazing group; MG group, mountain-grazing group.

**Table 7:** Volatile flavor compounds of LT muscle of goats in two groups.

Volatile Compound	Name	Relative Content (%)		SEM	*p*-Value
		FG Group	MG Group		
Aldehydes	Hexanal	6.25	9.55	0.96	0.009
	Heptanal	1.50	2.16	0.24	0.220
	(E)-2-Octenal	0.99	1.36	0.16	0.332
	Octanal	0.61	1.53	0.28	0.040
	Nonanal	9.87	10.06	0.11	0.491
	Decanal	5.15	5.82	0.39	0.510
	(E)-2-Nonenal	8.18	11.48	0.97	0.078
	10-Octadecenal	0.86	0.71	0.08	0.407
	Butanal-3-methyl	1.50	5.65	1.21	0.023
Alcohols	1-Pentanol	2.83	4.41	0.39	0.033
	1-Hexanol	2.00	3.66	0.52	0.079
	1-Octen-3-ol	16.48	13.67	0.71	0.006
	1-Octanol	9.39	14.83	1.10	0.001
	1-Heptanol	2.95	7.67	1.37	0.005
	2-Hexadecanol	2.74	1.44	0.39	0.049
	1-Hexanol-2-ethyl	1.61	5.03	1.00	0.013
	2,3-Butanediol	2.55	ND	-	NS
	3-Octyn-2-ol	ND	1.50	-	NS
	Z,Z-2,5-Pentadecadien-1-ol	2.10	0.64	0.44	0.051
Ketones	Acetone	2.47	2.56	0.13	NS
	2-Hexanone, 5-methy	0.23	ND	-	NS
Acids	Pentadecanoic acid	3.39	3.50	0.23	0.864
	Octadecanoic acid	4.28	ND	-	NS
Esters	Carbamodithioic acid, diethyl-, methyl ester	7.52	5.98	0.45	0.077
	Hexanoic acid, ethyl ester	3.52	4.13	0.19	0.079
	Phthalic acid, hexyl propyl ester	3.96	ND	-	NS
	Decanoic acid, octyl ester	3.57	ND	-	NS
Others	Toluene	1.15	1.50	0.11	0.047
	Ethylbenzene	2.10	2.55	0.21	0.382
	p-Xylene	2.49	2.55	0.13	0.863
	o-Xylene	3.63	3.60	0.17	0.984
	α-Muurolene	2.44	ND	-	NS
	Octadecane, 6-methyl	0.58	ND	-	NS
	Butane	1.24	ND	-	NS
	Ethanolamine	1.56	1.75	0.11	0.484
	Amphetamine	ND	1.70	-	NS
	Formamide, N,N-dibutyl	8.95	9.20	0.24	0.678

NS, not significant; ND, not detected. FG group, flatland-grazing group; MG group, mountain-grazing group.

**Table 8:** Relative odor activity value (ROAV) of the volatile flavor compounds of LT muscle of goats in two groups.

Volatile Compound	Threshold Value (ng/g)	Odor Descriptors	ROAV	
			FG Group	MG Group
Hexanal	10	Grassy	3.793	3.627
Heptanal	3	Jasmine, mint, burnt fat, green	3.039	2.739
(E)-2-Octenal	3	Wet ground, bitter, grass, meat, coffee	2.011	1.726
Octanal	0.7	Citrus-like, green, nutty, fatty	5.280	8.325
Nonanal	1	Rose fragrance	59.914	38.226
Decanal	0.1	Sweet Orange, Lemon, Rose, Wax	70.219	69.098
(E)-2-Nonenal	0.08	Fatty, tallow	92.022	100
1-Pentanol	4000	Fuel oil, fruit, balsamic	0.004	0.004
1-Hexanol	500	Woody, grass, fatty, fruity	0.024	0.028
1-Octen-3-ol	1	Mushroom, smoke	100	51.928
1-Octanol	126	Fatty, waxy, walnut, burnt	0.452	0.435
1-Heptanol	520	Fragrant, woody, green, fatty	0.034	0.055
1-Hexanol, 2-ethyl	270,000	Sweet, floral	<0.001	<0.001
Toluene	1550	Sweet	0.004	0.004
Ethylbenzene	2205.25	Sweet	0.006	0.004

FG group, flatland-grazing group; MG group, mountain-grazing group; ROAV, relative odor activity value.

## Data Availability

Not applicable.
